# Geographical distribution, genetic diversity, and environmental adaptations of dromedary camel breeds in Saudi Arabia

**DOI:** 10.3389/fvets.2024.1490186

**Published:** 2025-02-18

**Authors:** Mohanad A. Ibrahim, Marco Tolone, Mario Barbato, Faisal M. Alsubaie, Abdulwahed Fahad Alrefaei, Mikhlid Almutairi

**Affiliations:** ^1^Genalive Medical Laboratory, Riyadh, Saudi Arabia; ^2^Ministry of Environment, Water, and Agriculture (MEWA), Riyadh, Saudi Arabia; ^3^Data Science Program, King Abdullah International Medical Research Center, Riyadh, Saudi Arabia; ^4^Department of Chemical, Biological, Pharmaceutical, and Environmental Sciences, University of Messina, Messina, Italy; ^5^Department of Veterinary Science, Università degli Studi di Messina, Messina, Italy; ^6^Zoology Department, College of Science, King Saud University, Riyadh, Saudi Arabia; ^7^Genome Department, National Livestock and Fisheries Development Program, Riyadh, Saudi Arabia

**Keywords:** SNPs, dromedary camels, genetic diversity, Awarik, Majaheem, deserts

## Abstract

The dromedary camel (*Camelus dromedarius*) in Saudi Arabia exhibits significant genetic diversity, driven by adaptation to diverse ecological niches such as deserts, mountains, and coastal areas. This study explores the genetic structure of these camel populations, correlating their genetic diversity with geographical regions rather than ecological classifications. Through whole-genome sequencing of 63 camel genomes, we identified substantial differences in heterozygosity and inbreeding across different ecotypes, particularly noting higher genetic diversity in mountainous populations and lower diversity in coastal populations. The study also revealed significant enrichment of specific gene sets associated with environmental adaptation, such as the HECT domain in desert populations, which is crucial for maintaining protein integrity under extreme conditions. Principal component and admixture analyses further highlighted the genetic distinctiveness of certain breeds, particularly the Awarik (beach ecotype), which showed signs of genetic isolation.

## Introduction

The Arabian Peninsula is home to the dromedary camel (*Camelus dromedarius*), which has adapted to various ecological niches, including deserts, mountains, and coastal locations. This diversity of camel populations is noteworthy (1). Saudi Arabia provides an excellent environment for researching these camels’ genetic variety and adaptation techniques because of its varied settings (2). According to genetic studies, ecological categories do not correspond as well with the genetic structure of Saudi Arabian camels as geographic areas do. This suggests a complicated connection between genetic variety and environmental adaptability (2). Due to this genetic difference, different breeds in the north, middle, and western areas differ from those in the southwest and southeast (3).

A thorough analysis of the whole genome sequences of dromedaries from the Arabian Peninsula has shown a mostly uniform gene pool with few geographical variations (4). The genetic similarity observed across camel populations can be attributed to historical trade routes and transit activities facilitating gene flow between geographically distant regions. These trade networks allowed the movement and interbreeding of camels across diverse areas, reducing genetic differentiation. Additionally, the fact that camel owners do not have structured breeding programs has led to unmanaged breeding, which makes genetic traits even more similar. This lack of selective breeding aligns with observations by Al Abri and Faye (5), who noted that historic and ongoing movements of camels for trade and other purposes have significantly shaped their genetic landscape. Although there has been some genetic mixing, there has also been some geographic-associated structuring that has been identified, resulting in the division of camels into three main groups: those from the North, Central, and West and Southwest and Southeast regions of the Arabian Peninsula (6).

In the Arabian Peninsula and beyond, camels have been of paramount importance in the socio-economic progress of human civilizations (7). Primarily domesticated about 3,000–4,000 years ago in the southern Arabian Peninsula, dromedaries enabled transportation and commerce in dry areas, promoting cultural and economic interactions among far-flung populations (8). Camels offer milk, meat, and wool and are adaptable in challenging desert environments. Rendering them highly useful to nomadic and semi-nomadic communities (9). Moreover, camels retain cultural importance, frequently participating in customary festivities and competitions firmly established in Arabian cultures (10).

About 35 million dromedary camels exist worldwide, primarily in the arid and semi-arid regions of Australia, Asia, the Arabian Peninsula, and Africa. Food and Agriculture Organization (11) with around 1.6 million camels distributed throughout different areas and bred for racing, milk production, and meat, Saudi Arabia has a sizable camel population, which is indicative of its strong tradition in camel husbandry and breeding (2). The nation has fourteen recognized camel breeds, each suited to a specific type of climate. The north and central parts of the Arabian Peninsula are home to desert breeds such as Magaheem, Wodeh, Sofor, and Shual (12). While beach breeds like Sahlia and Awarik are located in the west and southwest coastal regions, mountain breeds like Hadana and Awadi live in the western and southwestern mountainous areas (13).

This study’s principal aim is to examine the genetic links across Saudi camel populations, particularly emphasizing groupings defined by geographical and environmental features, such as coastal, mountainous, and desert environments. To preserve and enhance this species, it is crucial to comprehend the genetic variety and organization of these many populations. For camel populations to be resilient and sustainable in the face of environmental and climatic difficulties, breeding plans should be based on insights from these kinds of studies (14).

## Materials and methods

### Sample collection

**Figure 1:**
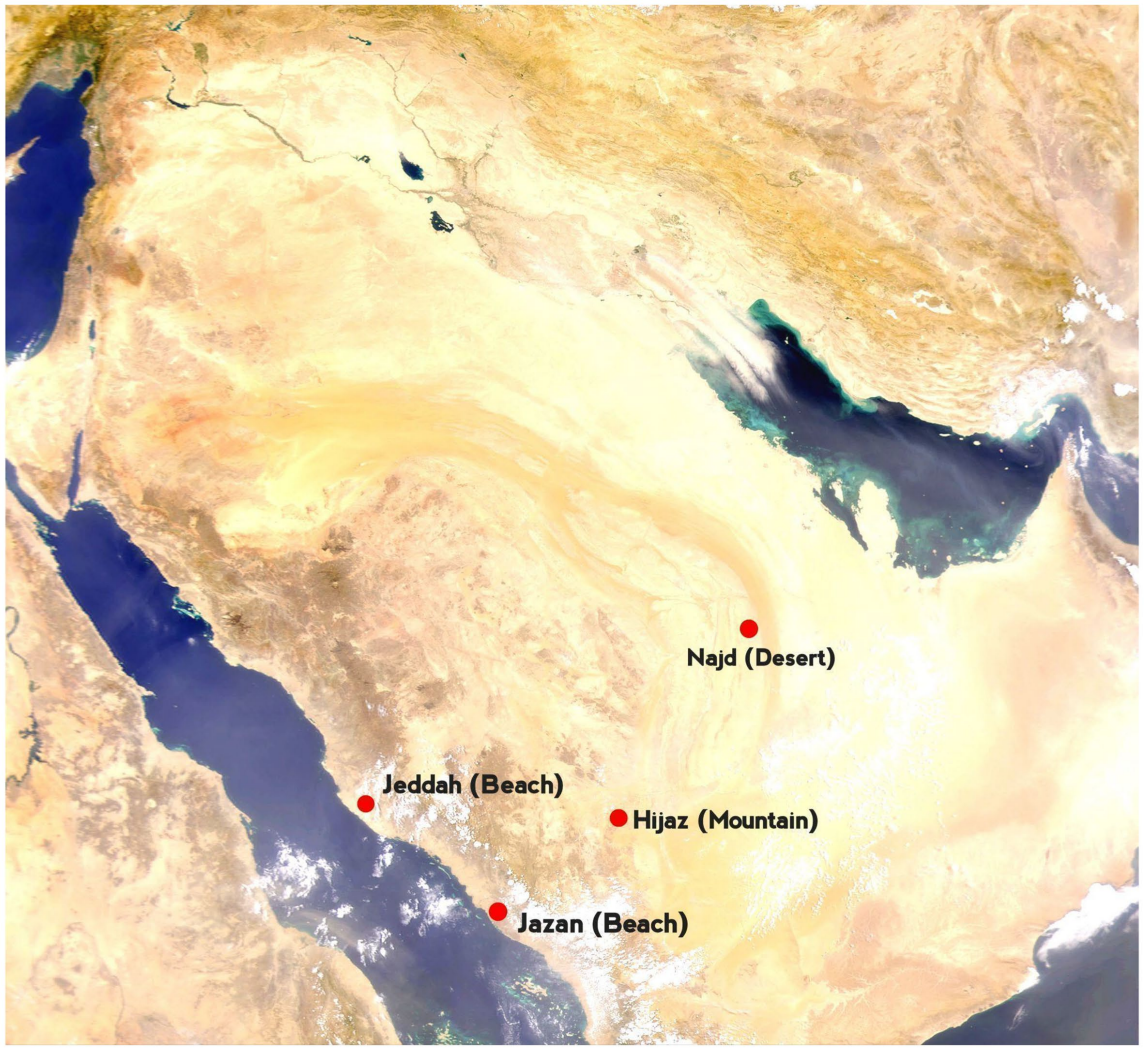
Map of Saudi Arabia. Red dots mark sampling locations. The labeled points represent key regions: Jeddah (Sahliah), Jazan (Awarik), Hijaz (Awadi, Haddana), and Najd (Majaheem, Shul, and Sofor).

The camels studied in this work were sampled from three distinct ecological environments: coastal, desert, and mountainous regions (Table 1, Figure 1). Based on their origins and breed names—historically documented and officially recorded by the Ministry of Agriculture and Environment—they were categorized into specific groupings. The coastal group included the “Sahliah” and “Awarik” breeds, primarily found near the Jeddah and South Jazan coastlines. The desert group comprised the “Sofor,” “Shul,” and “Majaheem” breeds, which have evolved to endure the harsh desert climates of Najad and Riyadh. Lastly, the “Awadi” and “Haddana” breeds, well-adapted to the rugged terrain of the Hijaz Mountains in southwestern Saudi Arabia, comprised the mountain group. Each breed represents a small, proportional part of the overall subpopulation within its respective ecological zone. Sampling was carried out on several farms, and individuals were selected based on information supplied by the farmers to avoid, as much as possible, closely related animals.

**Table 1:** Sampling distribution and characteristics across different habitat groups.

Group	Breed	Sample count	Sample location	Sex (M/F)	Age range
Beach	Sahliah	12	Coastal regions near Jeddah and South Jazan	8 M / 4F	Juvenile to Adult
	Awarik	9	Coastal regions near Jeddah and South Jazan	5 M / 4F	Juvenile to Adult
Deserts	Sofor	8	Najad and its eastern areas	4 M / 4F	Juvenile to Subadult
	Shul	10	Najad and its eastern areas	5 M / 5F	Subadult to Adult
	Majaheem	11	Najad and its eastern areas	6 M / 5F	Subadult to Adult
Mountain	Awadi	4	Hijaz mountains and southwestern KSA	2 M / 2F	Juvenile to Subadult
	Haddana	9	Hijaz mountains and southwestern KSA	5 M / 4F	Juvenile to Adult

The table summarizes the distribution of sampled camel breeds across three ecological groups (beach, deserts, and mountain), detailing sample count, geographic location, sex distribution (M/F), and age range.

### DNA extraction and sequencing

Following Wu et al. (15), we conducted blood collection and extracted genomic DNA using a Maxwell system (Promega, United States) with Maxwell RSC cartridges. We quantified the DNA concentration using the QuantiFluor dsDNA System and a Quantus Fluorometer and confirmed its purity and integrity through gel electrophoresis. Sampling was carried out on several farms, and individuals were selected based on information provided by farmers to minimize the inclusion of closely related animals. The Beijing Genomics Institute (BGI) performed whole-genome sequencing (WGS), fragmenting DNA into approximately 300 bp fragments using Covaris technology, followed by end repair, addition of an ‘A’ base to the 3′ ends, and adapter ligation. DNA nanoballs (DNBs) were generated from amplified libraries using rolling circle amplification (RCA), then loaded onto patterned nanoarrays and sequenced through the ILLUMINA NovaSeq 6,000 platform. High-throughput paired-end reads were generated with an average sequencing depth of 30× per genome. We evaluated the sequencing quality using FastQC v0.12.0 (16) (see Supplementary Table S1 for a detailed summary of the metrics).

### Data analysis

The sequencing workflow, as illustrated in Supplementary Figure S1, began with assessing data quality using FastQC v0.12.0 (16) to detect adapter contamination and low-quality nucleotides. Data cleaning was performed using SOAPnuke (17), configured for paired-end read cleaning. Reads were removed if they contained over 50% adapter sequences, more than 50% of bases with a Phred quality score below 20, or at least 2% ambiguous bases (“N”). Cleaned reads were aligned to the CamDro3 reference genome (NCBI Assembly)^1^ using BWA v0.7.18 (18), with the reference genome indexed using the bwa index command to enhance mapping efficiency. Alignments were stored in sequence alignment/map (SAM) format for subsequent analysis. Using SAMtools (19, 20), SAM files were converted into binary alignment/map (BAM) files sorted by genomic coordinates. Additional processing included marking shorter splits as secondary alignments (-M), enabling YML format output (-Y), and specifying read group information (-R) to ensure compatibility with downstream analyses.

Variant calling was performed using GATK HaplotypeCaller v4.5.0.0 (21) to identify high-confidence variants. The analysis applied specific criteria, including a minimum mapping quality of 20, a maximum of 6 alternative alleles, and a minimum base quality score of 20. These thresholds were based on the default recommendations from the GATK Best Practices pipeline (22). Variants were further filtered using stringent thresholds to exclude low-quality or potentially erroneous calls: quality by depth (QD) scores below 2.0, quality scores (QUAL) below 40.0, Fisher Strand (FS) values above 60.0, mapping quality (MQ) below 40.0, mapping quality rank sum (MQRankSum) below-12.5, and read position rank sum (ReadPosRankSum) below-8.0. These thresholds reflect widely accepted standards for distinguishing true variants from sequencing artifacts, as outlined in the GATK supported by Bahbahani et al. (23). To streamline processing, GVCF files generated for each sample were combined into a single consolidated file using GATK CombineGVCFs. The resulting file was then converted into VCF format using GATK GenotypeGVCFs, ensuring compatibility with downstream analyses, including those conducted using PLINK and other tools.

The discovered single nucleotide polymorphisms (SNPs) were refined for quality control using PLINK v1.9 software (24, 25). Variants with incomplete genotype data were eliminated by establishing a threshold to exclude SNPs with significant missing data (>0.05) with the --geno flag. A filter was subsequently applied based on Minor Allele Frequency (MAF) utilizing the --maf flag to select prevalent and informative SNPs by excluding less common variants (MAF <0.05). Linkage disequilibrium (LD) pruning was executed using the --indep-pairwise command with the settings (2000 k 1 0.5) to eliminate tightly connected SNPs. The selection of a 2000 kb window size and a r^2^ threshold of 0.5 was determined by camel genomics, characterized by large linkage disequilibrium blocks, in unlike humans. Similar techniques have been utilized in livestock species, including cattle, where bigger LD blocks necessitate appropriately sized windows (26). Furthermore, the --chr parameter focused exclusively on autosomal chromosomes, so excluding non-autosomal variants from influencing the results. The --rel-cutoff flag was employed to eliminate closely related samples, adhering to the relatedness estimation methodology established by Purcell et al. (25) and Manichaikul et al. (27) in PLINK.

To evaluate genetic diversity, the level of genetic variation within individuals was determined by calculating observed heterozygosity (H_o_) through a specific command (--het) in PLINK v1.9 (24, 25). This calculation assesses the overall genetic diversity by comparing the actual number of homozygous genotypes (O(HOM)) with the expected number (E(HOM)) in each individual, helping to estimate the inbreeding coefficient (F).

Runs of Homozygosity (ROH) were analyzed using the --homozyg command in PLINK. ROH detection relied on specific criteria, including the minimum number of SNPs, genomic density, and allowable gaps, to identify segments of homozygosity. ROHs were then categorized into three classes: small (<0.1 Mbp), medium (0.1–5 Mbp), and large (>5 Mbp), following thresholds widely used in livestock genetics (28, 29). These classification thresholds align with common patterns of genetic associations and inbreeding, making them suitable for camels given their genome size and linkage disequilibrium traits (26).

Effective population size (*N_e_*) was estimated with SNeP v1.11 (30), incorporating default settings and specific adjustments, including sample size correction for unphased genotypes, mutation rate correction, and the Sved and Feldman (31) mutation rate modifier.

The analysis of population structure involved the utilization of two complementary methodologies to gain a comprehensive understanding. Principal Component Analysis (PCA) was conducted using PLINK v1.9 to identify the main genetic variation trends among different populations. Furthermore, a model-based clustering method was employed with ADMIXTURE v1.3.0 (32) to ascertain the probable count of ancestral populations (K). ADMIXTURE was run for K values ranging from 2 to 5, with cross-validation to identify the optimal K based on prediction error.

Selection signatures were identified using the XP-nSL statistic implemented in Selscan v2.0.2 (33, 34) through pairwise comparisons between camel groups using phased data without setting the alternate flag. The analysis was configured with a scale parameter of 20,000 for normalizing extended haplotype homozygosity (EHH) decay curves, a maximum gap parameter of 200,000 to account for the camel genome’s larger LD blocks and recombination rates, and an EHH cutoff of 0.05 to reduce false positives while maintaining sensitivity. These parameters were chosen based on Selscan documentation and their effectiveness in species with similar genomic characteristics (35). To pinpoint important SNPs, XP-nSL scores were standardized, and variants with z-scores surpassing the critical value for a combined two-sided significance level of 0.05 were chosen. Extended selection windows were crafted by determining the average gap between adjacent SNPs and extending half of this distance on both sides to precisely depict genomic regions undergoing selection.

To annotate the selection windows, we used a reference GFF file to identify overlapping genes. The dataset was divided into three ecological groups and six populations. Genes were retained based on specific criteria to ensure robust and biologically meaningful results. Specifically, we isolated genes consistently under selection across all populations within an ecological group and retained only those present in every population of the group. Additionally, we extracted unique genes exclusive to one group but absent in the others, emphasizing ecological and population-specific adaptations. For functional analysis, significant Gene Ontology (G.O.) terms and pathways were identified using the Database for Annotation, Visualization, and Integrated Discovery (DAVID) (36), which provided insights into the biological functions and processes associated with the selected genes. These criteria ensured that the annotation process focused on genes with consistent and ecologically relevant selection signals.

## Results

### Data analysis

Sequencing of 63 camel genomes generated ~385 million reads (~57 billion bases). After applying quality refinement filters, including the removal of reads with >50% bases below a Phred quality score of 20, the dataset was consolidated to 373 million high-quality reads (~56 billion bases), resulting in a minimal data loss of 2.95%, which did not compromise downstream analyses. The refined dataset exhibited an average G.C. content of 42.40% and a Q30 score of 97.07% across all samples, indicating high sequencing accuracy and reliability (Supplementary Table S1).

Using BWA v0.7.18 for alignment, the reads were mapped to the CamDro3 reference genome, achieving an average mapping rate of 99.81% and a correctly paired read ratio of 99.69%, underscoring the dataset’s high reliability. The average sequencing depth was 21.5X, meaning the genome was covered more than five times in 92.87% of cases and at least one-fold in 93.74%. For more information, see Supplementary Table S2, which has detailed sequencing metrics for each sample.

Further analysis identified an average of 2,959,240 single nucleotide polymorphisms (SNPs) through variant calling, with a distribution of 2,870,314 homozygous SNPs and 88,927 heterozygous SNPs. This distribution underscores a significant prevalence of homozygous SNPs, constituting about 97.04% of the total SNPs, while heterozygous SNPs account for approximately 2.96%. Additionally, the transition-to-transversion ratio of nucleotide substitutions averaged 1.7027, highlighting the genetic variation within the sampled camel populations as depicted in Supplementary Table S3.

**Figure 2:**
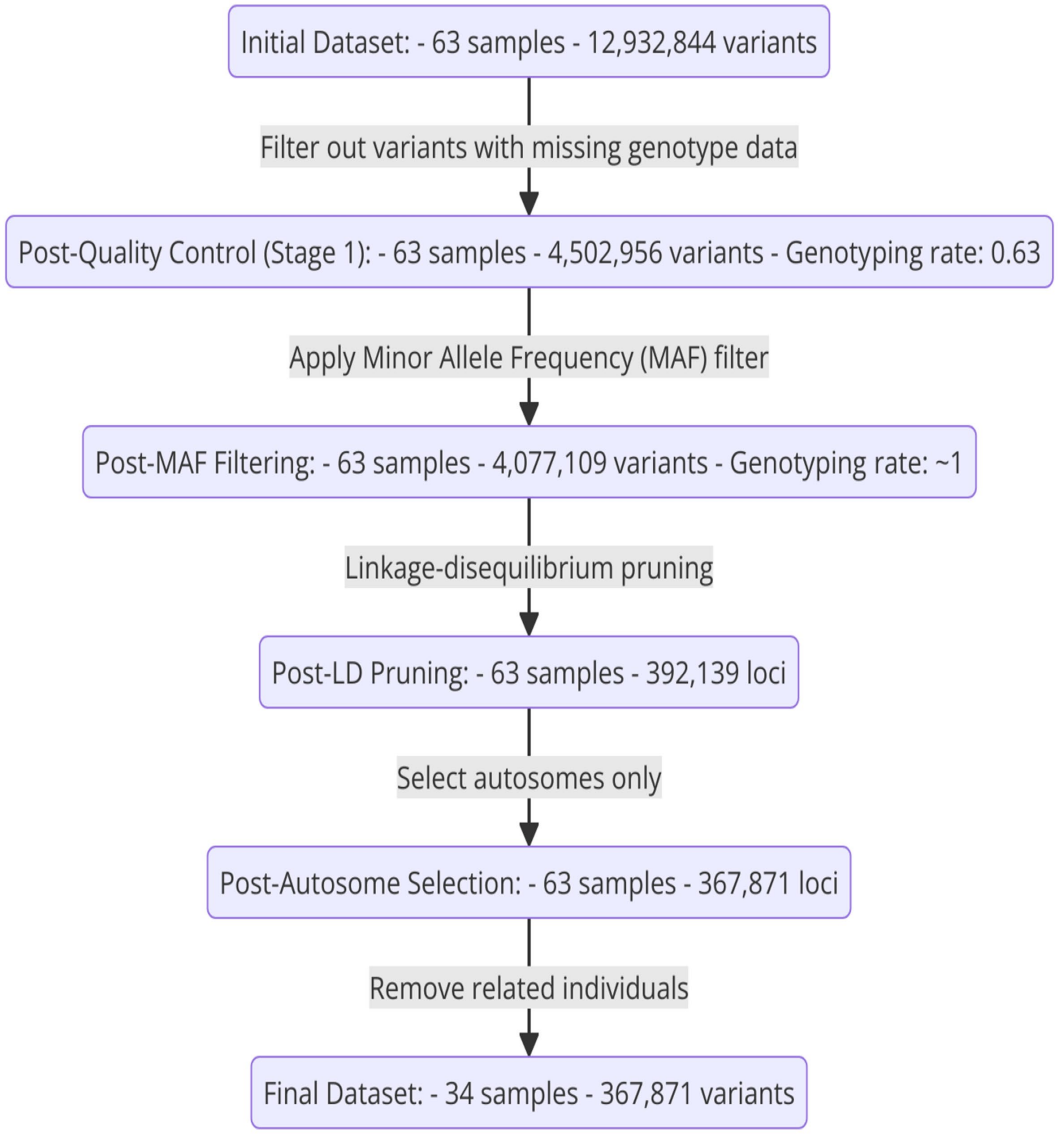
A diagram illustrating the sequential steps in the SNP filtering process, from the initial to the final refined dataset.

The initial dataset consisted of 63 samples and 12,932,844 variants. After the first stage of quality control, which involved filtering out variants with missing genotype data, the dataset was reduced to 4,502,956 variants, maintaining all 63 samples and achieving a genotyping rate of 0.63. Subsequent application of a minor allele frequency (MAF) filter further refined the dataset by removing 425,847 variants, resulting in 4,077,109 variants with a genotyping rate of ~1. Linkage disequilibrium pruning left 392,139 loci. Selecting autosomes exclusively further narrowed it down to 367,871 loci. Finally, removing related individuals led to a final dataset comprising 34 samples and 367,871 variants, ensuring high-quality data for subsequent analyses. In summary, see Figure 2.

### Genetic diversity

#### AWA

The H_o_ Table 2 across the populations ranges from 0.374 in the AWA population to 0.414 in the HAD population, indicating varying levels of genetic diversity. The HAD population shows the highest *H_o_* at 0.414, while the AWA population exhibits the lowest at 0.374. The other populations, including MAJ, SAH, SHU, and SOF, have H_o_ values between 0.396 and 0.405.

**Table 2:** Data on six camel populations including their acronyms, ecological groups (beach, mountain, and deserts), sample size *N*, observed heterozygosity H_o_ with standard deviation S.D., genomic inbreeding based on the proportion of the genome in runs of homozygosity *F_ROH_* with S.D.; and effective population size *N_e_*.

Population	Acronym	Ecotype	*N*	H_o_ (S.D.)	*F_ROH_* (S.D.)	*N_e_*
Awarik	AWA	Beach	5	0.374 (0.03)	0.015 (0.01)	15
Haddana	HAD	Mountain	4	0.414 (0.00)	0.003 (0.00)	11
Majaheem	MAJ	Deserts	9	0.400 (0.01)	0.002 (0.00)	37
Sahliah	SAH	Beach	7	0.405 (0.01)	0.000 (0.00)	24
Shul	SHU	Deserts	4	0.396 (0.01)	0.004 (0.01)	17
Sofor	SOF	Deserts	5	0.401 (0.01)	0.000 (0.00)	23

The effective population size *N_e_* varies significantly, with the MAJ population having the highest *N_e_* of 37, followed by SAH with 24 and SOF with 23. The HAD population has the smallest *N_e_* of 11, while the AWA and SHU populations have *N_e_* values of 15 and 17, respectively.

The *F_ROH_* values are generally low across all populations, with the AWA population having the highest value of 0.015 and the SAH and SOF populations showing the lowest values at 0.000. The remaining populations have values between 0.002 and 0.004.

**Figure 3:**
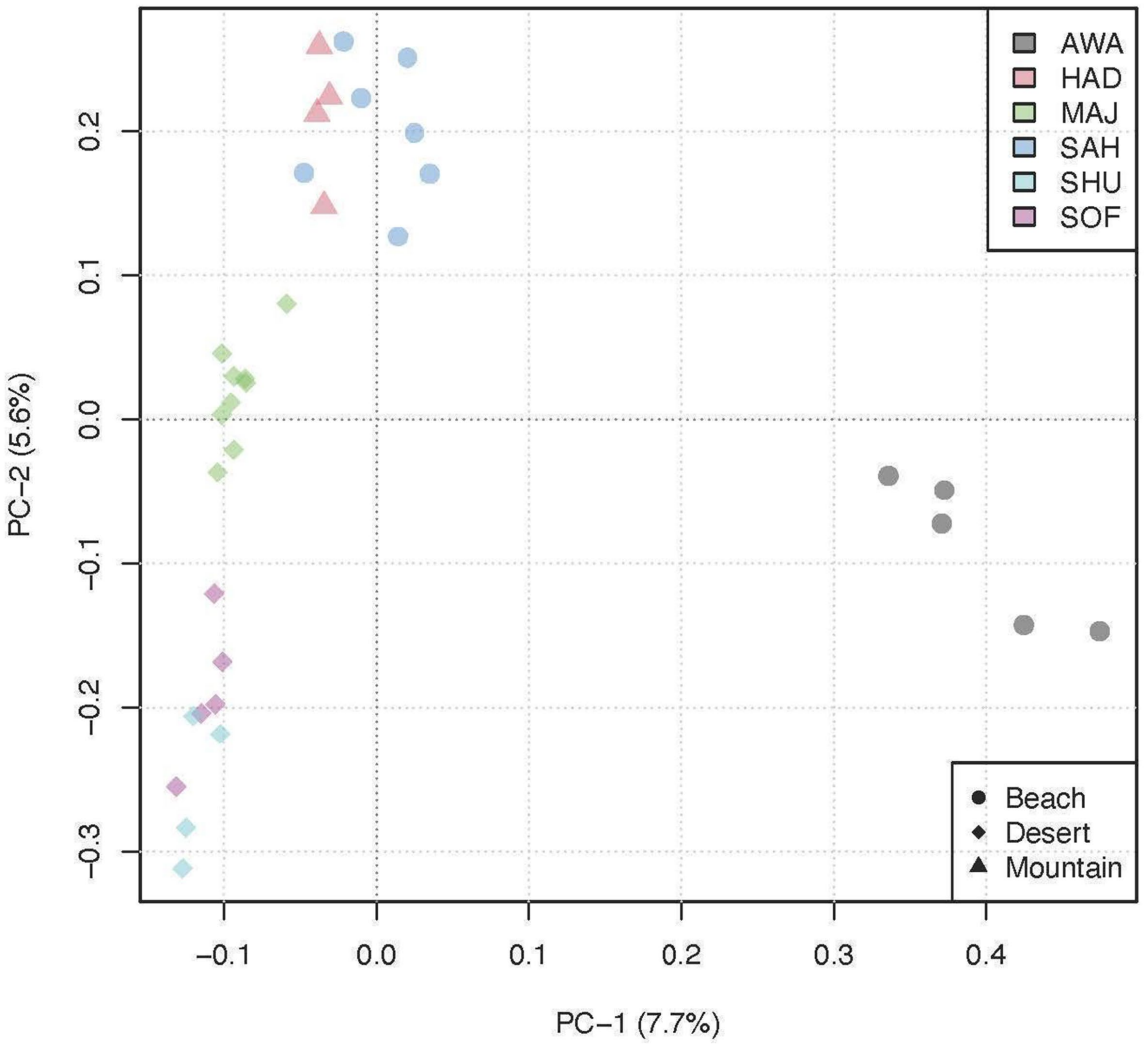
Principal Component Analysis (PCA) plot illustrating the genetic clustering of six camel breeds grouped into three environments.

The Principal Component Analysis (PCA) plot displays the genetic variation among the populations, with PC1 explaining 7.7% of the variance and PC2 explaining 5.6% (Figure 3). The plot reveals distinct clustering patterns: The desert populations (indicated by circles) are aligned along a single line on the right side of the plot, showing tight clustering along PC1. The mountain populations (indicated by triangles) cluster closely together along the positive side of PC2. In contrast, the beach populations (indicated by diamonds) are split into two groups: one group clusters closely with the mountain populations above, while the other group is positioned separately along the negative side of PC1, indicating distinct genetic differentiation within the beach ecotype.

**Figure 4:**
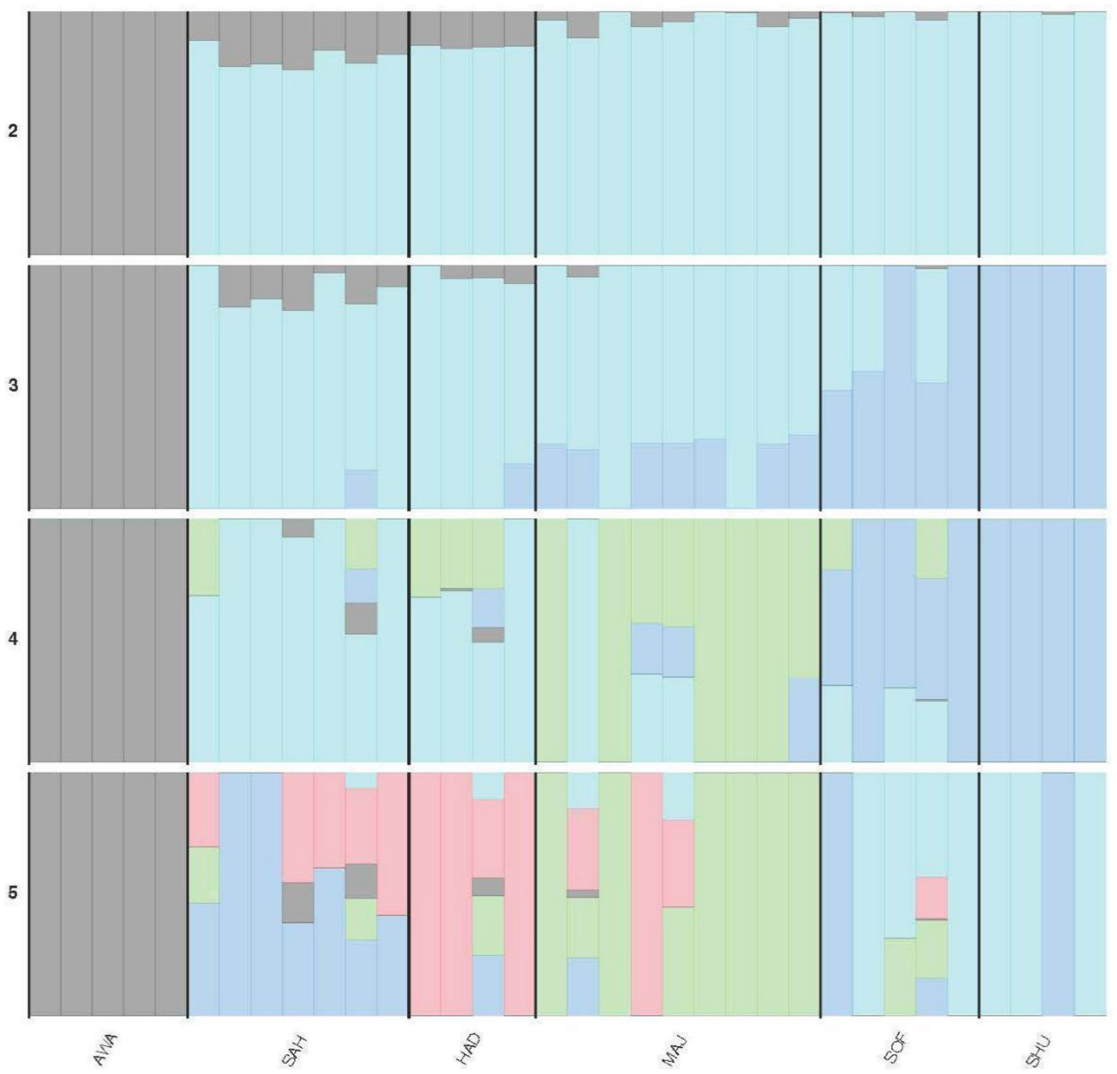
Admixture plot showing the genetic composition of six camel populations across K-values (K = 2 to 5). See Table 2 for population acronyms.

The admixture analysis for K values ranging from 2 to 5 reveals the following genetic structure among the populations (Figure 4). At the AWA, the population is distinctly separated from the others, with less than 10% of the AWA ancestral component appearing in SAH and HAD and a negligible presence in MAJ, SHU, and SOF. At K = 3, SHU acquires a private cluster is shared with SOF and partially with MAJ. At K = 4, a fourth ancestral cluster emerges, further diversifying HAD and MAJ. Finally, the genetic structure becomes more complex, with additional ancestral components present across all populations.

**Figure 5:**
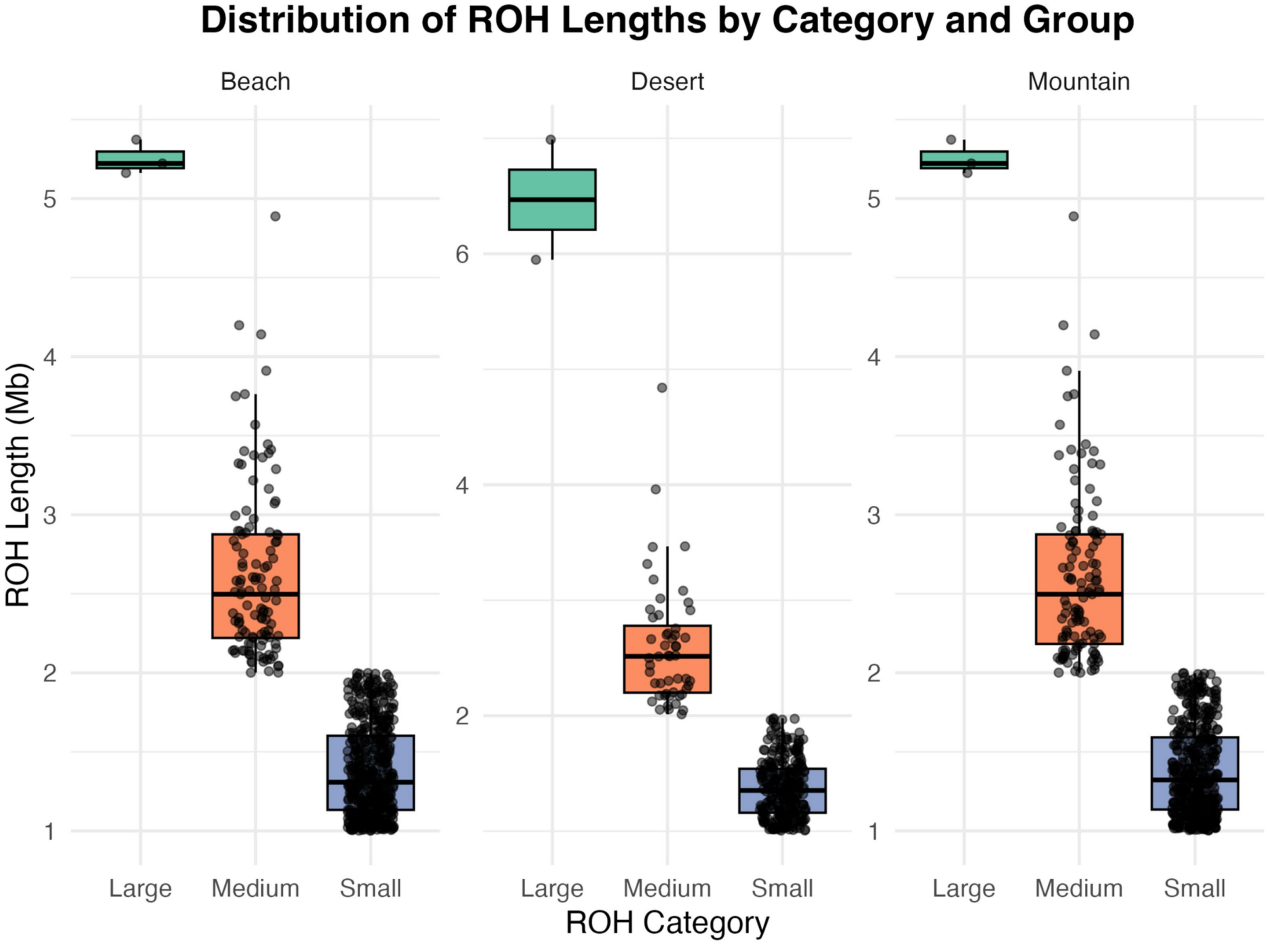
This box plot illustrates the distribution of Runs of Homozygosity (ROH) segment lengths across three categories—small, medium, and large—in different groups: beach, deserts, and mountain.

The analysis of ROH across different camel ecotypes reveals distinct patterns in the size categories of ROHs (Table 3; Figure 5)—classified as large (greater than 5 Mb), medium (2–5 Mb), and small (1–2 Mb). For the beach ecotype, the data shows 3 large ROHs totaling 15.76 Mb, 113 medium ROHs totaling 295.96 Mb, and 468 small ROHs with a total length of 646.34 Mb. In the desert ecotype, there are 2 large ROHs with a combined length of 12.94 Mb, 52 medium ROHs totaling 134.97 Mb, and 271 small ROHs accumulating to 371.00 Mb. The mountain ecotype exhibits three large ROHs. Totaling 15.76 Mb, 115 medium ROHs with a total length of 299.59 Mb, and 431 small ROHs summing to 595.75 Mb.

**Table 3:** Comparison of runs of homozygosity (ROH) categories across camel ecotypes.

ROH category	Beach (Count)	Deserts (Count)	Mountain (Count)
Large	3	2	3
Medium	113	52	115
Small	468	271	431

**Figure 6:**
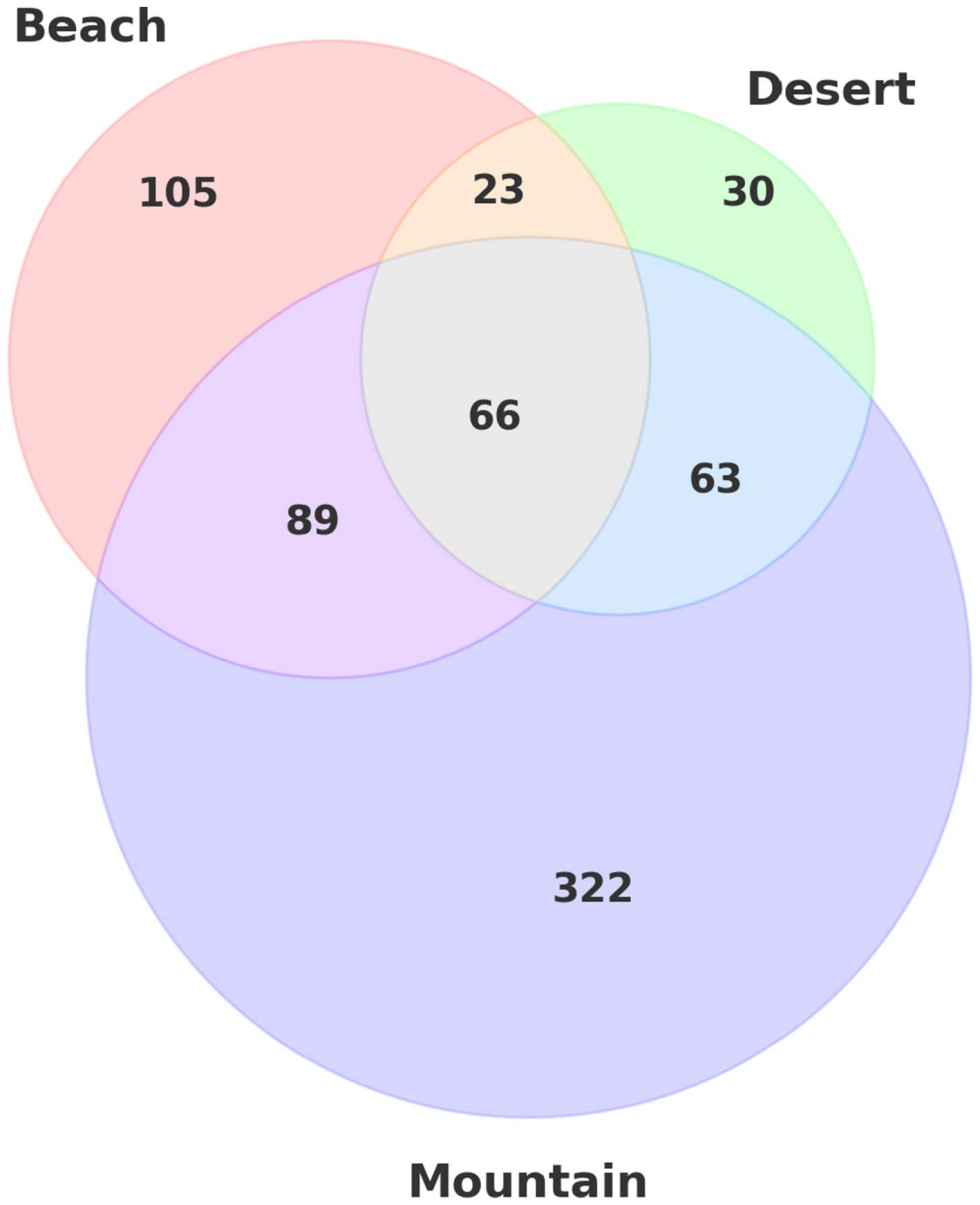
Venn diagram illustrating the distribution and intersection of unique gene sets across three different environmental groups: beach, deserts, and mountain.

Selection signatures were identified using the XP-nSL statistic, focusing on pairwise comparisons between camel groups. This method, which analyzes phased data to detect extended haplotype homozygosity, revealed genetic signals across the beach, deserts, and mountain camel populations. The results indicated that the beach group harbored 105 unique genes, the desert group had 30 unique genes, and the mountain group exhibited the most extensive selection, with 322 unique genes (Figure 6).

In addition to these unique signatures, there were also shared selection signals: 89 genes were common between the beach and mountain groups, 23 between the beach and desert groups, 63 between the desert and mountain groups, and 66 genes were shared across all three ecotypes.

The enrichment analysis, as detailed in Table 4, identified 21 genes in the deserts group with key functional annotations, including the HECT domain, basic and acidic residues, and disordered regions. This resulted in an enrichment score of 0.11 and significant *p*-values of 0.024 and 0.026. Table 5 highlights that the top enriched pathway in this group is Ubiquitin-ligase activity, with key genes such as HECTD4 and TMED5 noted in Table 6.

**Table 4:** The table summarizes the recognized genes, key functional annotations, enrichment scores, significant *p*-values, and example genes for the deserts, beach, and mountain groups.

Group	Recognized genes	Key functional annotations	Enrichment score (key cluster)	Significant *p*-values	Example genes
Deserts	21	HECT domain, basic and acidic residues, disordered	0.11	0.024 (HECT domain), 0.026 (HECT domain)	HECTD4, TMED5
Beach	90	P.H. domain, HECT domain, thyroid hormone synthesis	1.864	0.009 (P.H. domain), 0.030 (P.H. domain), 0.024 (HECT domain)	DAGLB, TMEM70
Mountain	274	B30.2/SPRY domain, HECT domain, transferase activity	2.655	0.000345 (B30.2/SPRY domain), 0.000410 (SPRY_dom), 0.024 (HECT domain)	ITGB1, KIF3B

**Table 5:** The top enriched pathways for the deserts, beach, and mountain groups and the corresponding *p*-values.

Group	Top enriched pathways	*p*-values
Deserts	Ubiquitin-ligase activity	0.024 (HECT domain), 0.026 (HECT domain)
Beach	Phosphatidylinositol signaling, thyroid hormone synthesis	0.009 (P.H. domain), 0.033 (Thyroid hormone synthesis), 0.024 (HECT domain)
Mountain	Immune response (B30.2/SPRY domains), metabolic adaptations	0.000345 (B30.2/SPRY domain), 0.000410 (SPRY domain), 0.024 (HECT domain)

**Table 6:** The table highlights specific genes, their functions, and their significance within the deserts, beach, and mountain ecotypes.

Group	Gene	Function	Significance
Deserts	*HECTD4*	Ubiquitin-ligase activity	Essential for protein maintenance in extreme temperatures
Beach	*TMED5*	Protein trafficking within the cell	Vital for maintaining cellular functions under stress.
	*DAGLB*	Endocannabinoid biosynthesis	Manages stress from varying salinity and nutrient availability
Mountain	*TMEM70*	Mitochondrial function and energy metabolism	Critical for adapting to the dynamic energy demands of coastal environments
	*ITGB1*	Cell adhesion and signal transduction	Adaptations to the physical demands of mountainous terrain
	*KIF3B*	Intracellular transport along microtubules	Supports cellular functions under high-altitude stress

In the beach group, as shown in Table 4, 90 genes were identified with key annotations, such as the P.H. domain, HECT domain, and thyroid hormone synthesis, yielding an enrichment score of 1.864. Significant p-values for these annotations include 0.009 and 0.030 for the P.H. domain and 0.024 for the HECT domain. Table 5 highlights that the top enriched pathways in this group are Phosphatidylinositol signaling and thyroid hormone synthesis, with example genes like DAGLB and TMEM70 listed in Table 6.

The mountain group, as detailed in Table 4, revealed 274 genes with key annotations, including the B30.2/SPRY domain, HECT domain, and transferase activity, achieving the highest enrichment score of 2.655. Significant p-values include 0.000345 for the B30.2/SPRY domain, 0.000410 for the SPRY domain, and 0.024 for the HECT domain. Table 5 indicates that the top enriched pathways in this group are related to immune response and metabolic adaptations, with critical genes such as ITGB1, KIF3B, and TMEM70 highlighted in Table 6.

## Discussion

Saudi Arabian dromedary camels vary genetically due to environmental constraints, breeding methods, and trading routes. These factors have changed camel genetics, improving their resilience and performance in harsh conditions. The genetic diversity of these populations is mostly uniform, although ecological and environmental changes have created geographic structure (6, 23, 37). Our dataset was identified in this study’s methodology. After alignment and purification, 99.81% of reads were mapped to the reference genome. After comprehensive quality control and filtering, 34 samples and 367,871 variations were left, ensuring high-quality data for additional investigations (Supplementary Tables S1–S3).

Our study revealed significant genetic patterns across different camel populations, highlighting variations in heterozygosity, effective population sizes, and selection signatures. Principal Component Analysis (PCA, Figure 3) showed a clear genetic separation between desert breeds (MAJ, SHU, and SOF), which clustered closely on the first two principal components (PC-1 and PC-2), and beach breeds (SAH and AWA), which formed distinct clusters. Notably, the AWA breed emerged as an independent cluster on PC-1, likely due to higher levels of inbreeding and genetic drift. This observation is supported by its low heterozygosity (H_o_ = 0.374) and the highest inbreeding coefficient (*F_ROH_* = 0.015) among all populations. These patterns may be influenced by geographic isolation or intentional breeding practices (6).

Admixture analysis (Figure 3) corroborates the PCA findings, revealing significant genetic mixing among breeds, such as SAH (Beach) and HAD (Mountain), suggesting historical gene flow or interbreeding. The K = 3 scenario revealed a desert-dependent genetic signature represented by the darker blue component, highlighting the unique genetic architecture of desert breeds. In contrast, the distinctiveness of AWA likely reflects its limited admixture and preservation of unique genetic traits, aligning with similar patterns observed in Omani dromedaries (23).

Runs of Homozygosity (ROH, Table 3; Figure 5) provided further insights into genetic architecture. Small ROHs (1–2 Mb), indicative of genetic drift and historical bottlenecks, dominated in the AWA population. Medium ROHs (2–5 Mb), linked to recent inbreeding or population substructure, were prevalent in HAD, while large ROHs (>5 Mb), indicative of very recent inbreeding, were distributed more evenly across beach and mountain populations but less so in desert breeds. These patterns reflect both ecological constraints and historical breeding practices.

The extremely low *N_e_* values across populations emphasize their vulnerability to genetic diversity loss, with the MAJ population demonstrating the highest *N_e_* (37), likely due to its broader distribution and higher gene flow. These findings align with previous research (6), emphasizing the role of historical admixture and environmental adaptation in shaping genetic structures.

Gene set enrichment and over-representation analyses highlight functional pathways critical for adaptation across ecotypes. For example, HECT family E3 ubiquitin ligases, prominent in desert camels, are pivotal for protein stability under oxidative stress, a condition prevalent in arid environments (38–43). Similarly, DAGLB and TMEM70, identified in beach ecotypes, play critical roles in lipid signaling and mitochondrial function, enabling adaptation to fluctuating salinity and nutrient availability (44–46). Immune response genes such as CX3CR1, IL6R, and CCR8, identified in desert camels, highlight adaptations to pathogen-rich environments (47, 48). Similarly, genes linked to fertility, such as ESR1 and SPACA5, emphasize the reproductive resilience of camels in harsh climates, paralleling findings in African zebu cattle (49, 50).

Energy metabolism genes, such as ESRRG and CRTC1, were identified as critical for energy homeostasis in desert and racing camels, underscoring the importance of efficient metabolic regulation for survival and stamina (51, 52). Chondrogenesis-related genes, including CHSY1 and CRLF1, reflect the physical demands placed on camels historically used for transport and racing, highlighting their role in skeletal adaptation (53, 54).

Genes associated with milk production (PICALM) and running performance (NAA16) further illustrate selective pressures shaped by cultural and economic practices (55, 56). These findings echo evolutionary pressures observed in cattle and other domesticated livestock, where production and performance traits have undergone significant selection.

Our findings highlight the intricate interplay between ecological and anthropogenic factors in shaping the genetic structure of camel populations. The distinct genetic profiles observed across ecotypes reflect adaptations to environmental pressures and historical breeding practices. Functional insights into key genes provide a deeper understanding of the mechanisms underlying resilience and performance in dromedary camels. These results, in line with prior studies (15, 23), underscore the importance of integrating genomic data with ecological and physiological studies to inform conservation and breeding strategies.

## Data Availability

The Genomic data have been deposited in NCBI repository, accession number PRJNA1219399. https://www.ncbi.nlm.nih.gov/bioproject/PRJNA1219399.

## Footnotes

^1^ https://www.ncbi.nlm.nih.gov/assembly/GCF_000803125.2
