# Supplementary material for: Geographical distribution, genetic diversity, and environmental adaptations of dromedary camel breeds in Saudi Arabia
Source: Front Vet Sci. 2025 Feb 18;11:1490186. doi: 10.3389/fvets.2024.1490186 (PMC11877447; doi:10.3389/fvets.2024.1490186)
Supplement: Supplementary file 1 [file Data_Sheet_1.docx]

Supplementary Material

Table S1: Quality Control Statistics

| **Sample** | **Raw read** | **Raw bases** | **Clean reads** | **Clean bases** | **Clean Data / Raw Data (%)** | **Clean GC rate(%)** | **Clean Q20(%)** | **Clean Q30(%)** |
| --- | --- | --- | --- | --- | --- | --- | --- | --- |
| **DM01** | 287460556 | 43119083400 | 279189622 | 41878443300 | 97.12 | 42.53 | 97.27 | 93.13 |
| **DM02** | 311419240 | 46712886000 | 301844990 | 45276748500 | 96.93 | 42.43 | 97.26 | 93.16 |
| **DM03** | 323440328 | 48516049200 | 315102480 | 47265372000 | 97.42 | 42.37 | 97.41 | 93.36 |
| **DM04** | 322310702 | 48346605300 | 313020292 | 46953043800 | 97.12 | 42.48 | 97.28 | 93.21 |
| **DM05** | 323431238 | 48514685700 | 315065956 | 47259893400 | 97.41 | 42.41 | 97.49 | 93.61 |
| **DM06** | 317496564 | 47624484600 | 306848766 | 46027314900 | 96.65 | 42.23 | 96.05 | 90.47 |
| **DM07** | 325071866 | 48760779900 | 315891830 | 47383774500 | 97.18 | 42.5 | 97.36 | 93.35 |
| **DM08** | 323437734 | 48515660100 | 314699972 | 47204995800 | 97.3 | 42.4 | 97.5 | 93.62 |
| **DM09** | 266092244 | 39913836600 | 258539672 | 38780950800 | 97.16 | 42.6 | 97.3 | 93.21 |
| **DM10** | 318432168 | 47764825200 | 309388138 | 46408220700 | 97.16 | 42.46 | 97.32 | 93.25 |
| **DM11** | 326705658 | 49005848700 | 316144250 | 47421637500 | 96.77 | 42.49 | 97.18 | 93 |
| **DM12** | 325074336 | 48761150400 | 315051714 | 47257757100 | 96.92 | 42.56 | 97.28 | 93.19 |
| **DM13** | 325073876 | 48761081400 | 316126290 | 47418943500 | 97.25 | 42.64 | 97.38 | 93.36 |
| **DM14** | 323440722 | 48516108300 | 314930140 | 47239521000 | 97.37 | 42.41 | 97.4 | 93.42 |
| **DM15** | 325980394 | 48897059100 | 315938014 | 47390702100 | 96.92 | 42.31 | 96.85 | 92.07 |
| **DM16** | 297895262 | 44684289300 | 289797498 | 43469624700 | 97.28 | 42.35 | 96.94 | 92.19 |
| **DM17** | 290231708 | 43534756200 | 281823042 | 42273456300 | 97.1 | 42.45 | 96.89 | 92.06 |
| **DM18** | 324342428 | 48651364200 | 315419316 | 47312897400 | 97.25 | 42.47 | 96.95 | 92.28 |
| **DM19** | 299644346 | 44946651900 | 291528398 | 43729259700 | 97.29 | 42.41 | 96.77 | 91.83 |
| **DM20** | 323438730 | 48515809500 | 315469132 | 47320369800 | 97.54 | 42.41 | 97.41 | 93.45 |
| **DM21** | 324342290 | 48651343500 | 314778406 | 47216760900 | 97.05 | 42.54 | 96.8 | 91.89 |
| **DM22** | 321141912 | 48171286800 | 312193564 | 46829034600 | 97.21 | 42.28 | 97.2 | 92.97 |
| **DM23** | 324341286 | 48651192900 | 315524242 | 47328636300 | 97.28 | 42.4 | 97.11 | 92.57 |
| **DM24** | 323436350 | 48515452500 | 314773468 | 47216020200 | 97.32 | 42.36 | 97.18 | 92.92 |
| **DM25** | 293335898 | 44000384700 | 285570102 | 42835515300 | 97.35 | 42.28 | 97.28 | 93.17 |
| **DM26** | 325070550 | 48760582500 | 316040110 | 47406016500 | 97.22 | 42.42 | 97.2 | 93.04 |
| **DM27** | 325072664 | 48760899600 | 315378816 | 47306822400 | 97.02 | 42.41 | 97.33 | 93.33 |
| **DM28** | 325073562 | 48761034300 | 315323634 | 47298545100 | 97 | 42.35 | 97.43 | 93.48 |
| **DM29** | 325070878 | 48760631700 | 315367838 | 47305175700 | 97.02 | 42.56 | 96.64 | 91.59 |
| **DM30** | 324342276 | 46651341786 | 315105766 | 47265864900 | 97.15 | 42.37 | 96.64 | 91.6 |
| **DM31** | 309554994 | 45633249658 | 300836502 | 45125475300 | 97.18 | 42.37 | 96.96 | 92.28 |
| **DM32** | 288925568 | 43338835200 | 273505632 | 41025844800 | 94.66 | 43.31 | 97.33 | 93.31 |
| **DM33** | 302050216 | 45307532365 | 291159762 | 43673964300 | 96.39 | 42.51 | 96.01 | 90.35 |
| **DM34** | 324341506 | 48651225900 | 315105264 | 47265789600 | 97.15 | 42.35 | 96.98 | 92.4 |
| **DM35** | 325072288 | 48760843200 | 315419960 | 47312994000 | 97.03 | 42.61 | 97.15 | 92.92 |
| **DM71** | 325072664 | 47651541437 | 313505622 | 47265789600 | 97.43 | 42.04 | 96.81 | 91.97 |
| **DM72** | 325073876 | 48151346696 | 313505695 | 47406016500 | 97.32 | 42.17 | 97.05 | 92.56 |
| **DM73** | 323438730 | 48651341400 | 293505697 | 47320369800 | 97.62 | 41.94 | 96.8 | 91.94 |
| **DM74** | 325073876 | 47651341432 | 303505684 | 47305175700 | 97.22 | 42.15 | 96.95 | 92.32 |
| **DM75** | 326705658 | 48651341498 | 313505952 | 47312897400 | 97.1 | 42.22 | 96.84 | 92.14 |
| **DR01** | 294919106 | 44237865900 | 284804620 | 42720693000 | 96.57 | 42.28 | 96.56 | 91.44 |
| **DR02** | 281163618 | 42174542700 | 272450740 | 40867611000 | 96.9 | 42.04 | 96.64 | 91.58 |
| **DR03** | 304648334 | 45697250100 | 293691492 | 44053723800 | 96.4 | 42.4 | 96.41 | 91.12 |
| **DR04** | 324341276 | 48651191400 | 314947046 | 47242056900 | 97.1 | 42.17 | 96.46 | 91.23 |
| **DR05** | 325978550 | 48896782500 | 315233730 | 47285059500 | 96.7 | 42.11 | 96.43 | 91.18 |
| **DR06** | 325979542 | 48896931300 | 315110654 | 47266598100 | 96.67 | 42.26 | 96.73 | 91.89 |
| **DR07** | 324340518 | 48651077700 | 315192188 | 47278828200 | 97.18 | 42.25 | 96.41 | 91.15 |
| **DR08** | 245721886 | 36858282900 | 238478372 | 35771755800 | 97.05 | 42.19 | 96.42 | 91.14 |
| **DR09** | 267770328 | 40165549200 | 259640242 | 38946036300 | 96.96 | 42.27 | 96.77 | 91.88 |
| **DR10** | 318670364 | 47800554600 | 310484440 | 46572666000 | 97.43 | 42.24 | 96.48 | 91.28 |
| **DR11** | 324340210 | 48651031500 | 314680516 | 47202077400 | 97.02 | 42.41 | 96.54 | 91.46 |
| **DR12** | 250275916 | 37541387400 | 243517996 | 36527699400 | 97.3 | 42.42 | 97.38 | 93.24 |
| **DR13** | 267636632 | 40145494800 | 259900234 | 38985035100 | 97.11 | 42.34 | 96.56 | 91.4 |
| **DR14** | 308394730 | 46259209500 | 299404536 | 44910680400 | 97.08 | 42.27 | 96.37 | 91.07 |
| **DR15** | 281862956 | 42279443400 | 272852862 | 40927929300 | 96.8 | 42.37 | 96.5 | 91.34 |
| **DR16** | 325073578 | 48761036700 | 315954702 | 47393205300 | 97.19 | 42.45 | 97.41 | 93.35 |
| **DR17** | 304059944 | 45608991600 | 296411620 | 44461743000 | 97.48 | 42.2 | 97.6 | 93.72 |
| **DR18** | 323435712 | 48515356800 | 314644516 | 47196677400 | 97.28 | 42.41 | 97.34 | 93.17 |
| **DR19** | 323440150 | 48516022500 | 314853882 | 47228082300 | 97.35 | 42.43 | 97.34 | 93.18 |
| **DR20** | 267504662 | 40125699300 | 259833066 | 38974959900 | 97.13 | 42.32 | 96.44 | 91.18 |
| **DR21** | 325072958 | 48760943700 | 316133062 | 47419959300 | 97.25 | 42.43 | 97.32 | 93.18 |
| **DR22** | 325070030 | 48760504500 | 315859530 | 47378929500 | 97.17 | 42.24 | 97.46 | 93.5 |
| **DR23** | 325071950 | 48760792500 | 316115676 | 47417351400 | 97.24 | 42.34 | 97.32 | 93.17 |
| **DR24** | 254624776 | 38193716400 | 247754542 | 37163181300 | 97.3 | 42.33 | 97.37 | 93.26 |
| **DR25** | 322822958 | 48423443700 | 314010786 | 47101617900 | 97.27 | 42.2 | 96.48 | 91.25 |
| **DR26** | 315759692 | 47363953800 | 307469550 | 46120432500 | 97.37 | 42.64 | 97.53 | 93.65 |
| **DR27** | 285437256 | 42815588400 | 277974806 | 41696220900 | 97.39 | 42.09 | 96.76 | 91.76 |
| **DR28** | 266505460 | 39975819000 | 257822156 | 38673323400 | 96.74 | 42.33 | 96.49 | 91.3 |
| **DR29** | 302921344 | 45438201600 | 295255080 | 44288262000 | 97.47 | 42.45 | 97.46 | 93.44 |
| **DR30** | 325978782 | 48896817300 | 315582402 | 47337360300 | 96.81 | 42.18 | 96.44 | 91.23 |
| **DR31** | 296573678 | 44486051700 | 288713502 | 43307025300 | 97.35 | 42.51 | 97.44 | 93.41 |
| **DR32** | 293960778 | 44094116700 | 286677120 | 43001568000 | 97.52 | 42.55 | 97.35 | 93.2 |
| **DR33** | 324199456 | 48629918400 | 313987970 | 47098195500 | 96.85 | 42.29 | 96.41 | 91.24 |
| **DR34** | 309015018 | 46352252700 | 299587944 | 44938191600 | 96.95 | 42.23 | 96.27 | 91 |
| **DR35** | 281519810 | 42227971500 | 271028540 | 40654281000 | 96.27 | 42.34 | 96.14 | 90.74 |
| **DR36** | 307299274 | 46094891100 | 299277160 | 44891574000 | 97.39 | 42.55 | 97.34 | 93.2 |
| **DR37** | 291224034 | 43683605100 | 283413546 | 42512031900 | 97.32 | 42.51 | 97.38 | 93.3 |
| **DR38** | 300528496 | 45079274400 | 292398144 | 43859721600 | 97.29 | 42.6 | 97.42 | 93.35 |
| **DR39** | 325073768 | 48761065200 | 315944978 | 47391746700 | 97.19 | 42.57 | 97.43 | 93.39 |
| **DR40** | 323430886 | 48514632900 | 315230844 | 47284626600 | 97.46 | 42.45 | 97.45 | 93.38 |

Table S2: Mapping Statistics

| **Samples** | **Clean reads** | **Mapping reads** | **Mapping rate** | **Properly paired reads** | **Properly paired ratio** | **Mean depth** | **Coverage >= 1X** | **Coverage >= 5X** | **Coverage >= 10X** | **Coverage >= 20X** | **Coverage >= 30X** | **Coverage >= 50X** |
| --- | --- | --- | --- | --- | --- | --- | --- | --- | --- | --- | --- | --- |
| **DM01** | 280651954 | 280102491 | 99.80% | 279718463 | 99.67% | 19.0548 | 93.71% | 92.59% | 85.28% | 30.85% | 3.79% | 0.83% |
| **DM02** | 303504757 | 302902358 | 99.80% | 302472110 | 99.66% | 20.588 | 93.74% | 92.80% | 87.51% | 38.26% | 5.44% | 0.90% |
| **DM03** | 316667297 | 316129524 | 99.83% | 315769968 | 99.72% | 21.5354 | 93.75% | 92.92% | 88.82% | 43.74% | 6.90% | 0.94% |
| **DM04** | 314693433 | 314037705 | 99.79% | 313585804 | 99.65% | 21.3526 | 93.76% | 92.88% | 88.30% | 41.97% | 6.56% | 0.92% |
| **DM05** | 316701802 | 316158790 | 99.83% | 315799949 | 99.72% | 21.5226 | 93.74% | 92.89% | 88.63% | 42.92% | 6.65% | 0.93% |
| **DM06** | 308575297 | 307791494 | 99.75% | 307179885 | 99.55% | 20.889 | 93.77% | 92.87% | 88.19% | 39.06% | 5.16% | 0.91% |
| **DM07** | 317604198 | 317021278 | 99.82% | 316605111 | 99.69% | 21.5582 | 93.74% | 92.87% | 88.57% | 42.92% | 6.76% | 0.96% |
| **DM08** | 316348107 | 315841576 | 99.84% | 315476370 | 99.72% | 21.5002 | 93.75% | 92.91% | 88.67% | 42.94% | 6.69% | 0.95% |
| **DM09** | 259909856 | 259444030 | 99.82% | 259105976 | 99.69% | 17.6475 | 93.71% | 92.17% | 80.99% | 22.85% | 2.62% | 0.77% |
| **DM10** | 311033444 | 310470343 | 99.82% | 310060663 | 99.69% | 21.1182 | 93.75% | 92.86% | 87.98% | 40.07% | 5.98% | 0.93% |
| **DM11** | 317941343 | 317246274 | 99.78% | 316771621 | 99.63% | 21.5489 | 93.74% | 92.87% | 88.57% | 42.74% | 6.60% | 0.91% |
| **DM12** | 316735572 | 315987616 | 99.76% | 315553733 | 99.63% | 21.4941 | 93.75% | 92.89% | 88.45% | 42.45% | 6.68% | 0.93% |
| **DM13** | 317807493 | 317240850 | 99.82% | 316859300 | 99.70% | 21.5882 | 93.75% | 92.88% | 88.28% | 42.09% | 6.76% | 0.95% |
| **DM14** | 316574213 | 316020061 | 99.82% | 315647661 | 99.71% | 21.5073 | 93.76% | 92.91% | 88.73% | 42.50% | 6.35% | 0.95% |
| **DM15** | 317552091 | 316952791 | 99.81% | 316604025 | 99.70% | 21.5808 | 93.74% | 92.81% | 87.68% | 41.85% | 7.15% | 0.92% |
| **DM16** | 291204768 | 290741126 | 99.84% | 290442713 | 99.74% | 19.8174 | 93.71% | 92.57% | 85.28% | 33.55% | 4.75% | 0.84% |
| **DM17** | 283208004 | 282742645 | 99.84% | 282432439 | 99.73% | 19.2645 | 93.71% | 92.49% | 84.31% | 30.65% | 4.08% | 0.84% |
| **DM18** | 317057626 | 316506859 | 99.83% | 316157470 | 99.72% | 21.5549 | 93.74% | 92.81% | 87.75% | 41.73% | 7.06% | 0.93% |
| **DM19** | 292963590 | 292483744 | 99.84% | 292180019 | 99.73% | 19.929 | 93.72% | 92.63% | 85.86% | 33.66% | 4.53% | 0.84% |
| **DM20** | 317120981 | 316494424 | 99.80% | 316061774 | 99.67% | 21.5409 | 93.75% | 92.92% | 88.88% | 45.76% | 8.08% | 0.94% |
| **DM21** | 316405758 | 315859909 | 99.83% | 315499115 | 99.71% | 21.5157 | 93.75% | 92.82% | 87.81% | 42.03% | 7.15% | 0.93% |
| **DM22** | 313780508 | 313153281 | 99.80% | 312696400 | 99.65% | 21.3 | 93.75% | 92.91% | 88.69% | 43.69% | 7.03% | 0.96% |
| **DM23** | 317099605 | 316596839 | 99.84% | 316300454 | 99.75% | 21.5784 | 93.72% | 92.82% | 88.06% | 42.28% | 6.98% | 0.92% |
| **DM24** | 316374731 | 315739781 | 99.80% | 315275135 | 99.65% | 21.4777 | 93.76% | 92.94% | 88.90% | 44.55% | 7.32% | 0.95% |
| **DM25** | 287072125 | 286453146 | 99.78% | 286019137 | 99.63% | 19.4822 | 93.72% | 92.71% | 86.62% | 34.95% | 4.58% | 0.86% |
| **DM26** | 317718899 | 317054459 | 99.79% | 316560266 | 99.64% | 21.5471 | 93.75% | 92.89% | 88.54% | 44.30% | 7.66% | 0.96% |
| **DM27** | 317104234 | 316417840 | 99.78% | 315934569 | 99.63% | 21.5064 | 93.73% | 92.87% | 88.47% | 44.14% | 7.65% | 0.95% |
| **DM28** | 316970058 | 316345326 | 99.80% | 315954134 | 99.68% | 21.527 | 93.74% | 92.88% | 88.47% | 43.81% | 7.43% | 0.95% |
| **DM29** | 316955687 | 316261416 | 99.78% | 315715013 | 99.61% | 21.5048 | 93.75% | 92.90% | 88.71% | 43.87% | 7.14% | 0.94% |
| **DM30** | 316623406 | 316012851 | 99.81% | 315629782 | 99.69% | 21.5273 | 93.75% | 92.88% | 88.36% | 42.14% | 6.55% | 0.93% |
| **DM31** | 302336192 | 301853436 | 99.84% | 301556585 | 99.74% | 20.5636 | 93.71% | 92.69% | 86.62% | 37.30% | 5.63% | 0.90% |
| **DM32** | 275164072 | 274658675 | 99.82% | 274283043 | 99.68% | 18.6602 | 93.66% | 91.99% | 81.17% | 28.66% | 4.56% | 0.81% |
| **DM33** | 292744746 | 292090863 | 99.78% | 291631942 | 99.62% | 19.8527 | 93.74% | 92.55% | 85.07% | 33.07% | 4.68% | 0.85% |
| **DM34** | 316791326 | 316208993 | 99.82% | 315838691 | 99.70% | 21.5164 | 93.75% | 92.82% | 87.69% | 41.36% | 6.90% | 0.94% |
| **DM35** | 317132052 | 316505413 | 99.80% | 316042696 | 99.66% | 21.5061 | 93.76% | 92.88% | 88.20% | 42.33% | 6.95% | 0.93% |
| **DM71** | 316701541 | 316102664 | 99.81% | 315783760 | 99.71% | 21.5419 | 93.74% | 92.82% | 87.87% | 42.51% | 7.32% | 0.96% |
| **DM72** | 316961620 | 316323273 | 99.80% | 315958524 | 99.68% | 21.5466 | 93.76% | 92.89% | 88.30% | 42.80% | 7.04% | 0.94% |
| **DM73** | 306655443 | 306155886 | 99.84% | 305836549 | 99.73% | 20.8643 | 93.75% | 92.80% | 87.47% | 39.57% | 6.07% | 0.92% |
| **DM74** | 283056837 | 282585018 | 99.83% | 282282307 | 99.73% | 19.2656 | 93.72% | 92.59% | 85.23% | 31.73% | 4.08% | 0.84% |
| **DM75** | 316473117 | 315925057 | 99.83% | 315588058 | 99.72% | 21.505 | 93.76% | 92.95% | 89.29% | 43.88% | 6.26% | 0.91% |
| **DR01** | 286256632 | 285757282 | 99.83% | 285451018 | 99.72% | 19.4541 | 93.72% | 92.66% | 85.83% | 31.73% | 3.81% | 0.86% |
| **DR02** | 273846026 | 273391293 | 99.83% | 273105855 | 99.73% | 18.6095 | 93.73% | 92.54% | 84.29% | 26.15% | 2.72% | 0.81% |
| **DR03** | 295202131 | 294659360 | 99.82% | 294306799 | 99.70% | 20.0494 | 93.75% | 92.73% | 86.48% | 33.96% | 4.30% | 0.87% |
| **DR04** | 316536660 | 315990507 | 99.83% | 315635465 | 99.72% | 21.5087 | 93.76% | 92.90% | 88.70% | 41.74% | 5.93% | 0.94% |
| **DR05** | 316835046 | 316249660 | 99.82% | 315900187 | 99.70% | 21.5229 | 93.77% | 92.93% | 88.90% | 42.75% | 6.18% | 0.93% |
| **DR06** | 316690147 | 316075892 | 99.81% | 315736024 | 99.70% | 21.511 | 93.76% | 92.91% | 88.73% | 41.94% | 5.94% | 0.92% |
| **DR07** | 316799850 | 316204714 | 99.81% | 315838123 | 99.70% | 21.518 | 93.76% | 92.89% | 88.42% | 42.12% | 6.45% | 0.95% |
| **DR08** | 239680316 | 239284198 | 99.83% | 239017703 | 99.72% | 16.2879 | 93.69% | 91.91% | 77.90% | 15.93% | 1.68% | 0.72% |
| **DR09** | 260976771 | 260570699 | 99.84% | 260307536 | 99.74% | 17.7444 | 93.70% | 92.29% | 81.80% | 22.38% | 2.36% | 0.78% |
| **DR10** | 312253308 | 311229748 | 99.67% | 310802490 | 99.54% | 21.1305 | 93.76% | 92.48% | 86.65% | 40.48% | 5.73% | 0.94% |
| **DR11** | 316475327 | 315455614 | 99.68% | 315020525 | 99.54% | 21.4108 | 93.76% | 92.54% | 87.12% | 41.98% | 5.95% | 0.93% |
| **DR12** | 244870155 | 244130506 | 99.70% | 243859164 | 99.59% | 16.5919 | 93.66% | 91.11% | 77.17% | 18.93% | 2.02% | 0.71% |
| **DR13** | 261371103 | 260568734 | 99.69% | 260223071 | 99.56% | 17.6946 | 93.70% | 91.56% | 80.03% | 23.02% | 2.53% | 0.79% |
| **DR14** | 300934228 | 300362555 | 99.81% | 299986562 | 99.69% | 20.4381 | 93.77% | 92.84% | 87.63% | 35.94% | 4.37% | 0.91% |
| **DR15** | 274246621 | 273754914 | 99.82% | 273419856 | 99.70% | 18.6231 | 93.73% | 92.49% | 83.95% | 26.77% | 2.95% | 0.81% |
| **DR16** | 317602916 | 317087648 | 99.84% | 316785982 | 99.74% | 21.5928 | 93.76% | 92.89% | 88.31% | 42.22% | 6.71% | 0.94% |
| **DR17** | 298016586 | 297161526 | 99.71% | 296834809 | 99.60% | 20.2106 | 93.72% | 92.30% | 85.48% | 37.34% | 5.28% | 0.88% |
| **DR18** | 316359196 | 315341100 | 99.68% | 314934417 | 99.55% | 21.4341 | 93.75% | 92.50% | 86.84% | 42.62% | 6.81% | 0.95% |
| **DR19** | 316598897 | 315646825 | 99.70% | 315264419 | 99.58% | 21.4506 | 93.76% | 92.47% | 86.67% | 42.08% | 6.71% | 0.98% |
| **DR20** | 261263722 | 260441866 | 99.69% | 260100148 | 99.55% | 17.6845 | 93.71% | 91.64% | 80.39% | 22.70% | 2.38% | 0.79% |
| **DR21** | 317916797 | 316904822 | 99.68% | 316514685 | 99.56% | 21.5324 | 93.75% | 92.51% | 86.94% | 43.68% | 7.35% | 0.96% |
| **DR22** | 317480900 | 316911131 | 99.82% | 316600261 | 99.72% | 21.5843 | 93.75% | 92.90% | 88.53% | 42.42% | 6.50% | 0.95% |
| **DR23** | 317706236 | 317188851 | 99.84% | 316882355 | 99.74% | 21.6036 | 93.75% | 92.86% | 88.11% | 42.42% | 7.01% | 0.95% |
| **DR24** | 249031188 | 248664419 | 99.85% | 248425231 | 99.76% | 16.9352 | 93.70% | 92.05% | 79.43% | 18.98% | 2.02% | 0.75% |
| **DR25** | 315546462 | 315002258 | 99.83% | 314645597 | 99.71% | 21.4418 | 93.75% | 92.89% | 88.55% | 41.66% | 5.99% | 0.92% |
| **DR26** | 309276255 | 308362557 | 99.70% | 308013841 | 99.59% | 20.9534 | 93.73% | 92.36% | 85.98% | 39.58% | 6.09% | 0.92% |
| **DR27** | 279354126 | 278906628 | 99.84% | 278617829 | 99.74% | 18.9959 | 93.73% | 92.61% | 85.27% | 28.94% | 3.15% | 0.81% |
| **DR28** | 259190926 | 258677473 | 99.80% | 258364429 | 99.68% | 17.6013 | 93.71% | 92.24% | 81.36% | 21.95% | 2.33% | 0.76% |
| **DR29** | 296839180 | 296390609 | 99.85% | 296111098 | 99.75% | 20.1874 | 93.74% | 92.74% | 86.57% | 35.27% | 4.80% | 0.87% |
| **DR30** | 317377250 | 316295335 | 99.66% | 315815402 | 99.51% | 21.4634 | 93.77% | 92.55% | 87.13% | 42.98% | 6.53% | 0.93% |
| **DR31** | 290264708 | 289808086 | 99.84% | 289516459 | 99.74% | 19.7376 | 93.74% | 92.71% | 86.24% | 33.26% | 4.23% | 0.88% |
| **DR32** | 288235462 | 287776048 | 99.84% | 287496748 | 99.74% | 19.5957 | 93.75% | 92.67% | 85.69% | 31.58% | 3.90% | 0.85% |
| **DR33** | 315823027 | 314746815 | 99.66% | 314211318 | 99.49% | 21.3248 | 93.75% | 92.49% | 86.80% | 42.19% | 6.54% | 0.94% |
| **DR34** | 301343814 | 300243087 | 99.63% | 299675536 | 99.45% | 20.3389 | 93.76% | 92.40% | 85.96% | 36.67% | 4.56% | 0.88% |
| **DR35** | 272646633 | 271534093 | 99.59% | 270973865 | 99.39% | 18.3812 | 93.73% | 91.91% | 82.44% | 26.19% | 2.77% | 0.82% |
| **DR36** | 300842453 | 300351563 | 99.84% | 300038996 | 99.73% | 20.4577 | 93.75% | 92.79% | 87.22% | 36.65% | 4.97% | 0.87% |
| **DR37** | 284896556 | 284448515 | 99.84% | 284168717 | 99.74% | 19.3694 | 93.73% | 92.64% | 85.46% | 31.30% | 3.86% | 0.84% |
| **DR38** | 293892487 | 293479760 | 99.86% | 293225894 | 99.77% | 19.9951 | 93.73% | 92.71% | 86.46% | 34.80% | 4.66% | 0.87% |
| **DR39** | 317583230 | 317082430 | 99.84% | 316771903 | 99.74% | 21.6017 | 93.74% | 92.89% | 88.68% | 43.42% | 6.93% | 0.92% |
| **DR40** | 316989059 | 316031303 | 99.70% | 315654825 | 99.58% | 21.4844 | 93.76% | 92.54% | 87.09% | 43.38% | 6.84% | 0.95% |

Table S3: SNP Statistics

| **Samples** | **SNP numbers** | **Homo SNP number** | **Hete SNP number** | **Homo SNP rate(%)** | **Hete SNP rate(%)** | **Ts** | **Tv** | **Ts/Tv** |
| --- | --- | --- | --- | --- | --- | --- | --- | --- |
| **DM01** | 2915658 | 2831186 | 84472 | 97.1 | 2.9 | 1866703 | 1084137 | 1.7218 |
| **DM02** | 2971845 | 2884341 | 87504 | 97.06 | 2.94 | 1896897 | 1112357 | 1.7053 |
| **DM03** | 2972021 | 2886116 | 85905 | 97.11 | 2.89 | 1897693 | 1109135 | 1.711 |
| **DM04** | 2990706 | 2902440 | 88266 | 97.05 | 2.95 | 1909187 | 1118850 | 1.7064 |
| **DM05** | 2974992 | 2887113 | 87879 | 97.05 | 2.95 | 1899511 | 1111873 | 1.7084 |
| **DM06** | 2935261 | 2847582 | 87679 | 97.01 | 2.99 | 1872899 | 1101440 | 1.7004 |
| **DM07** | 2992090 | 2903903 | 88187 | 97.05 | 2.95 | 1908404 | 1120732 | 1.7028 |
| **DM08** | 3004999 | 2916268 | 88731 | 97.05 | 2.95 | 1916923 | 1124803 | 1.7042 |
| **DM09** | 2929261 | 2842574 | 86687 | 97.04 | 2.96 | 1870298 | 1097315 | 1.7044 |
| **DM10** | 2979167 | 2890471 | 88696 | 97.02 | 2.98 | 1898035 | 1118570 | 1.6968 |
| **DM11** | 2869387 | 2781487 | 87900 | 96.94 | 3.06 | 1823884 | 1083189 | 1.6838 |
| **DM12** | 2955757 | 2868013 | 87744 | 97.03 | 2.97 | 1883134 | 1109572 | 1.6972 |
| **DM13** | 2991455 | 2901320 | 90135 | 96.99 | 3.01 | 1902579 | 1127618 | 1.6873 |
| **DM14** | 2906875 | 2819464 | 87411 | 96.99 | 3.01 | 1848138 | 1095269 | 1.6874 |
| **DM15** | 2895383 | 2809717 | 85666 | 97.04 | 2.96 | 1848336 | 1083967 | 1.7052 |
| **DM16** | 2930565 | 2842655 | 87910 | 97 | 3 | 1872785 | 1097344 | 1.7067 |
| **DM17** | 2912219 | 2826045 | 86174 | 97.04 | 2.96 | 1858795 | 1091248 | 1.7034 |
| **DM18** | 2941882 | 2855415 | 86467 | 97.06 | 2.94 | 1880000 | 1099027 | 1.7106 |
| **DM19** | 2937561 | 2851029 | 86532 | 97.05 | 2.95 | 1875468 | 1099918 | 1.7051 |
| **DM20** | 2967997 | 2880392 | 87605 | 97.05 | 2.95 | 1893330 | 1111299 | 1.7037 |
| **DM21** | 2963578 | 2875978 | 87600 | 97.04 | 2.96 | 1893050 | 1108977 | 1.707 |
| **DM22** | 2924465 | 2837912 | 86553 | 97.04 | 2.96 | 1862642 | 1097029 | 1.6979 |
| **DM23** | 2953506 | 2866933 | 86573 | 97.07 | 2.93 | 1886093 | 1103691 | 1.7089 |
| **DM24** | 2966748 | 2879758 | 86990 | 97.07 | 2.93 | 1893174 | 1109256 | 1.7067 |
| **DM25** | 2958562 | 2873930 | 84632 | 97.14 | 2.86 | 1893702 | 1099404 | 1.7225 |
| **DM26** | 2945176 | 2858945 | 86231 | 97.07 | 2.93 | 1877763 | 1103095 | 1.7023 |
| **DM27** | 2920339 | 2833228 | 87111 | 97.02 | 2.98 | 1862255 | 1094757 | 1.7011 |
| **DM28** | 2974326 | 2885319 | 89007 | 97.01 | 2.99 | 1896925 | 1115604 | 1.7004 |
| **DM29** | 2883550 | 2798849 | 84701 | 97.06 | 2.94 | 1840366 | 1078542 | 1.7063 |
| **DM30** | 2935933 | 2850308 | 85625 | 97.08 | 2.92 | 1874395 | 1097253 | 1.7083 |
| **DM31** | 2912525 | 2827587 | 84938 | 97.08 | 2.92 | 1861733 | 1086680 | 1.7132 |
| **DM32** | 2804420 | 2718315 | 86105 | 96.93 | 3.07 | 1788494 | 1055610 | 1.6943 |
| **DM33** | 2797268 | 2712968 | 84300 | 96.99 | 3.01 | 1782023 | 1053283 | 1.6919 |
| **DM34** | 2968653 | 2880382 | 88271 | 97.03 | 2.97 | 1891297 | 1115424 | 1.6956 |
| **DM35** | 2977940 | 2891143 | 86797 | 97.09 | 2.91 | 1902078 | 1111579 | 1.7111 |
| **DM71** | 3080831 | 2986597 | 94234 | 96.94 | 3.06 | 1950924 | 1165406 | 1.674 |
| **DM72** | 3090287 | 2997722 | 92565 | 97 | 3 | 1957087 | 1166259 | 1.6781 |
| **DM73** | 3086039 | 2993786 | 92253 | 97.01 | 2.99 | 1957708 | 1161406 | 1.6856 |
| **DM74** | 3061104 | 2968297 | 92807 | 96.97 | 3.03 | 1939449 | 1156092 | 1.6776 |
| **DM75** | 3042209 | 2951875 | 90334 | 97.03 | 2.97 | 1927441 | 1146513 | 1.6811 |
| **DR01** | 2930896 | 2846450 | 84446 | 97.12 | 2.88 | 1876847 | 1090284 | 1.7214 |
| **DR02** | 2914520 | 2829615 | 84905 | 97.09 | 2.91 | 1864885 | 1086489 | 1.7164 |
| **DR03** | 2940711 | 2853056 | 87655 | 97.02 | 2.98 | 1874732 | 1104628 | 1.6972 |
| **DR04** | 2958822 | 2870792 | 88030 | 97.02 | 2.98 | 1886715 | 1110394 | 1.6991 |
| **DR05** | 2979460 | 2892805 | 86655 | 97.09 | 2.91 | 1902895 | 1112605 | 1.7103 |
| **DR06** | 2996493 | 2908529 | 87964 | 97.06 | 2.94 | 1911488 | 1122262 | 1.7032 |
| **DR07** | 2975351 | 2887799 | 87552 | 97.06 | 2.94 | 1897049 | 1116270 | 1.6995 |
| **DR08** | 2879302 | 2794961 | 84341 | 97.07 | 2.93 | 1842764 | 1074941 | 1.7143 |
| **DR09** | 2919891 | 2834365 | 85526 | 97.07 | 2.93 | 1867851 | 1090365 | 1.7131 |
| **DR10** | 2994456 | 2907987 | 86469 | 97.11 | 2.89 | 1910668 | 1119721 | 1.7064 |
| **DR11** | 2985622 | 2897250 | 88372 | 97.04 | 2.96 | 1903655 | 1119354 | 1.7007 |
| **DR12** | 2921643 | 2835154 | 86489 | 97.04 | 2.96 | 1869076 | 1092021 | 1.7116 |
| **DR13** | 2947487 | 2859402 | 88085 | 97.01 | 2.99 | 1883203 | 1105221 | 1.7039 |
| **DR14** | 2946462 | 2860552 | 85910 | 97.08 | 2.92 | 1879931 | 1102806 | 1.7047 |
| **DR15** | 2928299 | 2842535 | 85764 | 97.07 | 2.93 | 1873731 | 1093157 | 1.7141 |
| **DR16** | 2982095 | 2893121 | 88974 | 97.02 | 2.98 | 1902924 | 1117057 | 1.7035 |
| **DR17** | 2970171 | 2880713 | 89458 | 96.99 | 3.01 | 1893867 | 1115190 | 1.6982 |
| **DR18** | 2988694 | 2900670 | 88024 | 97.05 | 2.95 | 1904839 | 1120407 | 1.7001 |
| **DR19** | 3015984 | 2926899 | 89085 | 97.05 | 2.95 | 1922646 | 1130854 | 1.7002 |
| **DR20** | 2950485 | 2863741 | 86744 | 97.06 | 2.94 | 1885303 | 1103951 | 1.7078 |
| **DR21** | 3009302 | 2920875 | 88427 | 97.06 | 2.94 | 1916036 | 1129880 | 1.6958 |
| **DR22** | 2933294 | 2847178 | 86116 | 97.06 | 2.94 | 1871099 | 1097282 | 1.7052 |
| **DR23** | 2976049 | 2888045 | 88004 | 97.04 | 2.96 | 1897725 | 1115646 | 1.701 |
| **DR24** | 2942405 | 2855986 | 86419 | 97.06 | 2.94 | 1879532 | 1101319 | 1.7066 |
| **DR25** | 2955256 | 2867912 | 87344 | 97.04 | 2.96 | 1885176 | 1107669 | 1.7019 |
| **DR26** | 2986367 | 2897117 | 89250 | 97.01 | 2.99 | 1903537 | 1120735 | 1.6985 |
| **DR27** | 2935940 | 2849509 | 86431 | 97.06 | 2.94 | 1875039 | 1098650 | 1.7067 |
| **DR28** | 2926601 | 2840317 | 86284 | 97.05 | 2.95 | 1872858 | 1093703 | 1.7124 |
| **DR29** | 2958318 | 2870842 | 87476 | 97.04 | 2.96 | 1888424 | 1107146 | 1.7057 |
| **DR30** | 2989904 | 2901243 | 88661 | 97.03 | 2.97 | 1904714 | 1123314 | 1.6956 |
| **DR31** | 2961421 | 2874775 | 86646 | 97.07 | 2.93 | 1889761 | 1107920 | 1.7057 |
| **DR32** | 2977889 | 2890112 | 87777 | 97.05 | 2.95 | 1899042 | 1115567 | 1.7023 |
| **DR33** | 2987224 | 2897644 | 89580 | 97 | 3 | 1905255 | 1121794 | 1.6984 |
| **DR34** | 2984774 | 2896253 | 88521 | 97.03 | 2.97 | 1900813 | 1122302 | 1.6937 |
| **DR35** | 2955288 | 2868768 | 86520 | 97.07 | 2.93 | 1890171 | 1103827 | 1.7124 |
| **DR36** | 2948038 | 2860887 | 87151 | 97.04 | 2.96 | 1879263 | 1104701 | 1.7012 |
| **DR37** | 2962029 | 2875705 | 86324 | 97.09 | 2.91 | 1892556 | 1105841 | 1.7114 |
| **DR38** | 2950223 | 2862673 | 87550 | 97.03 | 2.97 | 1883355 | 1104496 | 1.7052 |
| **DR39** | 2938524 | 2852217 | 86307 | 97.06 | 2.94 | 1874627 | 1099176 | 1.7055 |
| **DR40** | 2991015 | 2903272 | 87743 | 97.07 | 2.93 | 1909535 | 1117382 | 1.7089 |


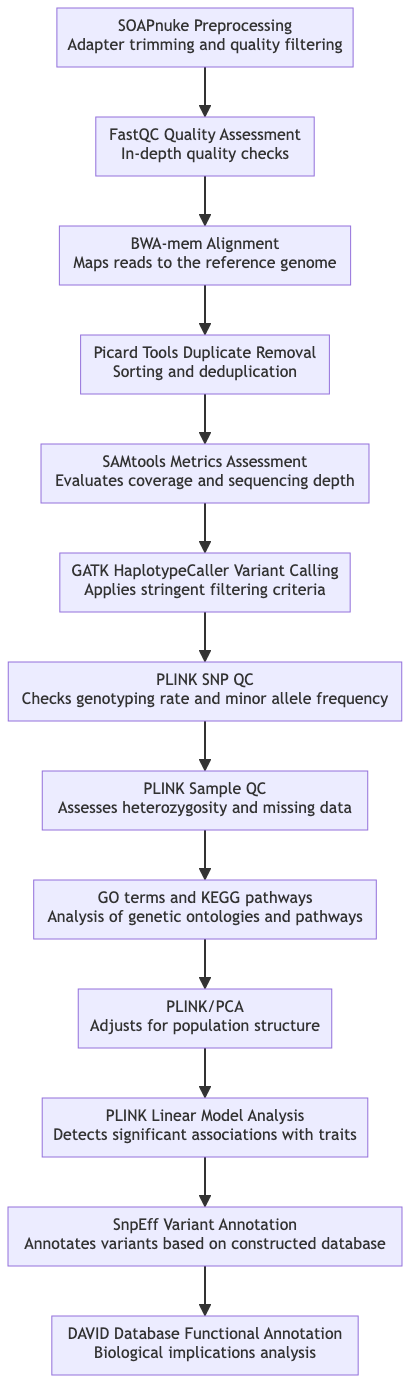


Figure. S1 outlines the comprehensive genomics data processing pipeline, showcasing each critical step from raw data preprocessing to functional annotation. This sequence of operations ensures data accuracy for the subsequent analysis phases.


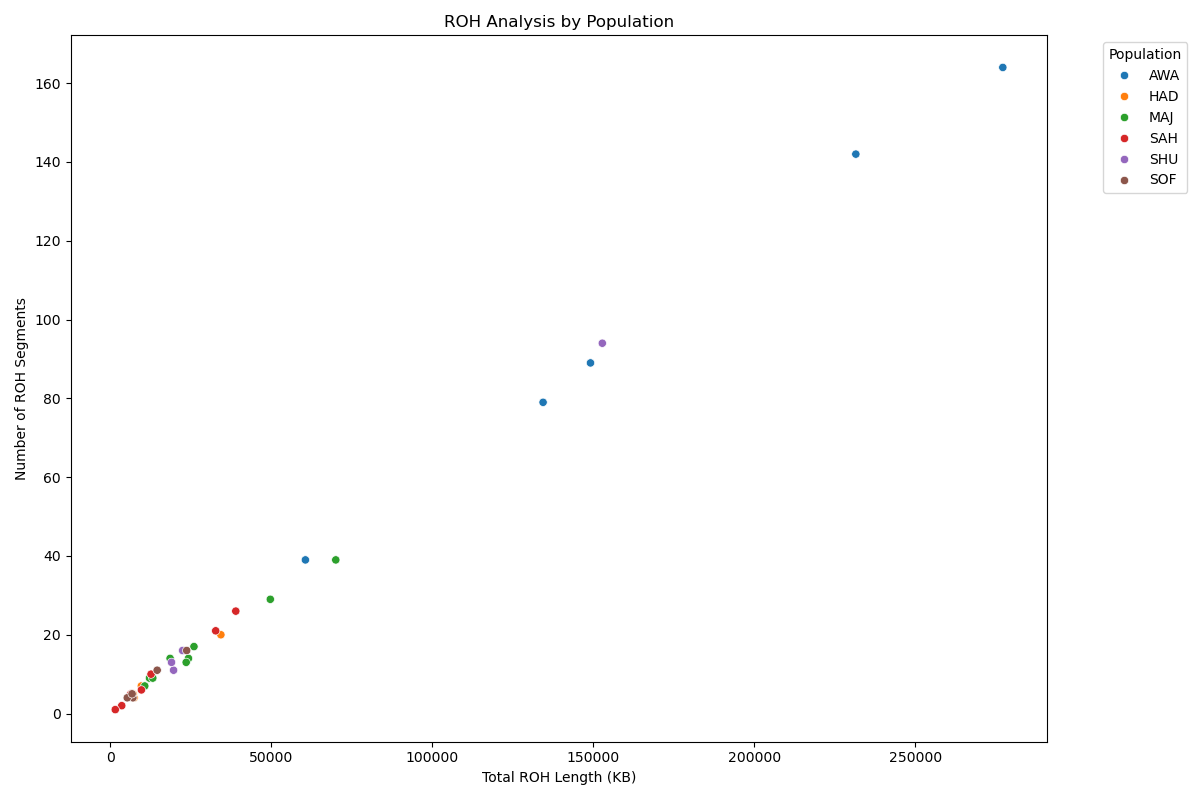


Figure S2: Number of ROH by length for each individual within camel populations
